# Violence at work experienced by nursing professionals working in hospital units: an exploratory and correlational study*

**DOI:** 10.1590/1518-8345.7451.4527

**Published:** 2025-03-31

**Authors:** Eduarda dos Santos Amaral, Gisele Arruda, Alessandro Rodrigues Perondi, Jolana Cristina Cavalheiri, Ana Paula Vieira, Franciele Ani Caovilla Follador

**Affiliations:** 1 Universidade Estadual do Oeste do Paraná, Centro de Ciências da Saúde, Francisco Beltrão, PR, Brazil; 2 Scholarship holder at the Coordenação de Aperfeiçoamento de Pessoal de Nível Superior (CAPES), Brazil; 3 Universidade Paranaense, Curso de Enfermagem, Francisco Beltrão, PR, Brazil

**Keywords:** Workplace Violence, Nursing, Exposure to Violence, Social Behavior, Working Conditions, Occupational Health

## Abstract

to identify the occurrence of violence at work affecting nursing professionals working in hospitals and to relate professional profile variables to this phenomenon.

this is an exploratory, cross-sectional, descriptive, correlational, field and quantitative study carried out with 218 nursing professionals working in hospital units in the 8th Health Region of Paraná, using a sociodemographic questionnaire and the Questionnaire for the Evaluation of Violence at Work Suffered or Witnessed by Nursing Workers. Data was analyzed using absolute and relative frequencies, and the Chi-squared test with Yates’ continuity correction was used to verify the associated factors.

the sample included 218 nursing professionals, 44.0% of whom reported having suffered violence at work, 11.9% physical violence, 47.7% verbal abuse and 2.8% sexual harassment. When the association was made, it was observed that professionals over 30 and who work overtime suffer more violence than other professionals.

in view of the above, it was possible to see a significant occurrence of episodes of violence at work in the last 12 months, with verbal violence being the most frequently reported.

## Highlights

**(1)** Significant prevalence of violence in the workplace. **(2)** Emphasis on workers’ health for nursing professionals. **(3)** Verbal violence affects 47.7% of the sample studied. **(4)** The need for public policies to improve safety at work. **(5)** Encouragement for further research on the subject.

## Introduction

Work is a constant presence in society and is essential for personal and professional development, as well as for economic and social progress ^(1)^. However, with the advance of new technologies, work activities have demanded ever more knowledge and commitment from professionals, which has led to overload and wear and tear on individuals ^(2)^.

In addition, the stress and agitation of everyday life can lead to various comorbidities for professionals, which directly interfere with the population’s quality of life ^(3)^. Among the factors that trigger psychosomatic illnesses, violence in the workplace stands out as one of the main causes of work-related illness ^(4)^.

Healthcare workers experience violence in the workplace in all its aspects (general, physical, verbal and sexual), especially nurses, because they are in direct contact with patients and their families for a long time, are the front line of healthcare, and are present at times of pain, anguish, suffering and death, making the team susceptible to violence ^(5)^.

The occurrence of violence can cause damage to the institution and to workers’ health, with the consequences for the institutional sphere being absenteeism and presenteeism, a decrease in organizational commitment and a reduction in the quality of the work provided. For the worker, it can lead to psychological repercussions such as Burnout Syndrome and minor psychological disorders, physical and social injuries, violation of personal integrity, rights and dignity ^(6)^.

In addition, repeated exposure to violence leads to the development of physiological and psychological responses, such as depression, anxiety, sleep disorders and isolation, often prompting the need for medication, which can reduce productivity and the quality of care provided ^(7)^.

A study carried out by the Regional Nursing Council of São Paulo ^(8)^ between 2015 and 2017, with 8,332 correspondents, showed that 74% of professionals had suffered violence in the workplace, and 73% said that the incidents continued to happen. A survey carried out in the southern region of Brazil found that 63% of participants had been victims of violence in the last 12 months, with psychological violence being the most frequently reported (48.7%) ^(9)^.

In this way, the development of this study will contribute to an assessment of the violence suffered by professionals working in hospital units, making it possible to recognize the gaps that make it difficult to reduce these acts and to institute actions that can help reduce cases. As such, it will enable the development of public policies aimed at workers’ health, given that there are not enough studies on the subject in the southwest region of Paraná. In addition, studies on institutional violence can contribute to the development of future research, enable critical reflection on the phenomenon of violence in society and how it affects health services, as well as providing the basis for specific labor legislation for health professionals.

Based on this premise, the aim of this study was to identify the occurrence of violence at work affecting nursing professionals working in hospitals and to relate professional profile variables to this phenomenon.

## Method

### Type of study

This is an exploratory, cross-sectional, descriptive, correlational and quantitative study carried out with nursing professionals working in hospital units.

### Study environment

The research was carried out in the state of Paraná, Brazil, which has a population of 11,443,208 inhabitants, according to data from the last census carried out in 2022 ^(10)^. The Southwest region, where the study was carried out, covers 27 municipalities in Paraná, subdivided into 13 small, medium and large hospitals that provide services of lesser complexity or urgency and emergency, in different specialties, all via the Unified Health System - SUS.

### Study participants

The study population was made up of all the nursing professionals working in the SUS hospitals of the 8th Health Region of Paraná, who were invited to take part in the study, totaling 744 workers. Of this total, 218 nursing professionals took part in the study, 91 nurses (41.7%) and 127 nursing technicians (58.3%).

The sample size was calculated according to the Open Source Epidemiologic Statistics for Public Health application (2022) ^(11)^, which indicated the need for at least 209 participants in order to achieve a power of 80% and a significance level of 0.05 or less. For this calculation, we used data from recent literature in Brazil, according to which the prevalence of violence at work is 40% ^(8 - 14)^. Thus, a population (N) of 744 individuals (563 nurse technicians; 181 nurses) in 11 municipalities, totaling 13 hospital units for data collection, was estimated in the calculation. Therefore, using an average prevalence of 40% (±5%) in people (p) in a model in which the limits of the confidence interval are 95% (d) with a design effect (DE) of 1 (random sample).

### Inclusion and exclusion criteria

Nursing professionals who had been working in the institution for at least 30 days were invited to take part in the study, excluding those who were not working in the unit at the time stipulated by the researchers, as well as professionals who were on vacation.

### Data collection instrument

Data collection took place between February and August 2023, using self-administered questionnaires via an online form, the aim of which was to identify the profile of the professionals. The questionnaires were prepared by the researchers using data from the literature, investigating the variables of age, gender, schooling, skin color, marital status, professional category, length of time working at the institution, management position, whether they had more than one employment relationship, work shift and whether they had ever suffered violence at work.

The second instrument was a questionnaire validated in Brazil, based on the model of the World Health Organization, the International Labor and Public Services Organization and the International Council of Nursing, entitled Questionnaire for the Evaluation of Violence at Work Suffered or Witnessed by Nursing Workers ^(15)^. The questionnaire consists of 54 questions, divided into physical violence in the workplace (17 questions), verbal abuse in the workplace (16 questions), sexual harassment in the workplace (16 questions), other types of violence in the workplace reported by the worker (3 questions), prevention and reduction of violence in the workplace (3 questions), covering the episodes suffered, the gender of the aggressor, forms of prevention, etc ^(15)^.

### Data analysis

Before analysis, variables were checked and recoded. Absolute and relative frequencies were used to describe the information. The Chi-square test with Yates’ continuity correction was used to verify the factors associated with the different types of violence at work. The analyses were carried out using the SPSS 29.0 ^(16)^ program, assuming a significance level of p <0.05.

### Ethical procedures

As this is research involving human beings, the study was first submitted to the Research Ethics Committee, and authorization was sought with informed consent from the institutions chosen as the study environment, prior to data collection. Once both had given their consent, data was collected from the selected sample, after they had accepted the Informed Consent Form (ICF). The data was collected using an online form sent to the participants via electronic means (such as WhatsApp, email, for example).

The ethical and legal aspects essential for scientific research were preserved, as well as the secrecy and confidentiality of the participants’ data, respecting Resolution 466/2012 of the National Health Council. The project was submitted to the Ethics Committee and approved under opinion number 5.623.032.

## Results

Table 1 shows the main characteristics of the sample. It can be seen that the majority of participants were female, over 30 years old, white-skinned, nursing technicians, with no managerial positions. With regard to types of violence, verbal violence predominated (47.7%), while physical violence (11.9%) and sexual abuse (2.8%) were less commonly reported. Approximately 11.0% of the sample reported having chronic illnesses.

**Table 1 -:** Characterization of the sample (n = 218). Southwest Paraná, PR, Brazil, 2023

	**n**	**%**
**Sex**		
Female	177	81.2
Male	41	18.8
**Education**		
Complete technical education	109	50.0
Higher education incomplete	19	8.7
Higher education complete	90	41.3
**Age**		
20 to 30 years	75	34.4
Over 30 years old	143	65.6
**Marital status**		
Single	90	41.3
Married	116	53.2
Divorced/Widowed	12	5.5
**Skin color**		
White	162	74.3
Brown	49	22.5
Black	7	3.2
**Category**		
Nurse	91	41.7
Nursing Technician	127	58.3
**Do you work overtime?**		
Yes	86	39.4
No	132	60.6
**Do you have a managerial position?**		
Yes	28	12.8
No	190	87.2
**Work shift**		
Daytime	119	54.6
Evening	69	31.7
Both	30	13.8
**Have you suffered violence in the workplace?**		
Yes	96	44.0
No	122	56.0
**Have you suffered physical violence at work?**		
Yes	26	11.9
No	192	88.1
**Have you suffered verbal violence at work?**		
Yes	104	47.7
No	114	52.3
**Have you suffered sexual harassment at work?**		
Yes	6	2.8
No	210	97.2

The factors associated with the different types of violence are shown in Table 2. Statistically significant differences (p <0.05%) were found in relation to working overtime, which indicated that participants who reported working overtime had a higher prevalence of general violence, verbal violence and sexual abuse. There were descriptive differences, which indicated that there was a higher frequency of general violence among men, but physical violence was more frequent among women. Descriptively, the highest incidences of violence were observed among older people (verbal and general) and among divorced/widowed people (general and sexual).

**Table 2 -:** Factors associated with violence among nursing professionals. Southwest Paraná, PR, Brazil, 2023

**Exhibitions**	**General violence**	**Physical violence**	**Verbal violence**	**Sexual abuse**
	**%**	**%**	**%**	**%**
**Sex**				
Female	40.7	13.0	45.8	2.8
Male	58.5	7.3	56.1	2.6
p	0.057	0.457	0.308	0.929
**Education**				
Complete technical education	40.4	14.7	45.0	4.6
Higher education incomplete	36.8	0.0	36.8	0.0
Higher education completed	50.0	11.1	53.3	1.1
p	0.318	0.181	0.305	0.245
**Age**				
20 - 30 years	41.3	8.0	48.0	1.4
Over 30 years old	45.5	14.0	47.6	3.5
p	0.661	0.195	0.950	0.357
**Marital status**				
Single	48.9	8.9	52.2	1.1
Married	39.7	12.9	42.2	4.3
Divorced/widowed	50.0	25.0	66.7	0.0
p	0.380	0.240	0.146	0.318
**Skin color**				
White	42.0	9.9	45.1	3.1
Brown	51.0	20.4	59.2	2.1
Black	42.9	0.0	28.6	0.0
p	0.534	0.070	0.128	0.760
**Category**				
Nurse	50.5	9.9	53.8	2.2
Nursing Technician	39.4	13.4	43.3	3.2
p	0.133	0.566	0.162	0.675
**Do you work overtime?**				
Yes	**54.7**	14.0	**65.1**	**6.0**
No	**37.1**	10.6	**36.4**	**0.8**
p	**0.016**	0.595	**<0.001**	**0.024**
**Do you have a managerial position?**				
Yes	46.4	17.9	50.0	3.7
No	43.7	11.1	47.4	2.6
p	0.785	0.469	0.795	0.754
**Work shift**				
Daytime	47.1	12.6	51.3	2.6
Evening	39.1	7.2	43.5	1.4
Both	43.3	20.0	43.3	6.7
p	0.571	0.187	0.515	0.341

Table 3 shows the profile of the aggressors and the consequences reported for each type of violence. It can be seen that men are the main aggressors, both for physical violence and sexual abuse. Patients were the main aggressors for physical violence, while bosses/superiors were the main aggressors for sexual abuse. In terms of consequences, the most commonly reported impairments were anxiety, disappointment, stress, irritation and loss of job satisfaction.

**Table 3 -:** Profile of aggressors and consequences of each type of violence. Southwest Paraná, PR, Brazil, 2023

**Exhibitions**	**Physical violence**	**Verbal violence**	**Sexual abuse**
	**%**	**%**	**%**
**Aggressor**			
Patient	74.1	51.0*	33.3
Patient’s relative	25.9	46.2	0.0
Boss/superior	0.0	33.7	66.7
**Sex of the aggressor**			
Female	22.2	41.6	0.0
Male	77.8	58.4	100
Consequences*			
Time off work	0.0	2.0	0.0
Anxiety	22.2	37.0	33.3
Low self-esteem	29.6	29.0	33.3
Fatigue	18.5	22.0	0.0
Crying spells	29.6	17.0	33.3
Disappointment	59.3	46.0	33.3
Difficulty sleeping	14.8	13.0	33.3
Pain	11.1	1.0	16.7
Stress	33.3	70.0	100
Irritation	40.7	58.0	83.3
Bodily injury	0.0	0.0	0.0
Fear	11.1	12.0	16.7
Loss of concentration	7.4	13.0	50.0
Loss of job satisfaction	14.8	40.0	83.3
Anger	14.8	27.0	66.7
Feeling of inferiority	11.1	34.0	83.3
Sadness	37.0	36.0	50.0

*Separate questions, so they add up to more than 100%

## Discussion

Nursing professionals play a crucial role in maintaining, improving and enhancing people’s health awareness, as well as being fundamental in hospital care and other health services ^(17)^. On the other hand, due to the multiple demands, high workload and shift work, healthcare workers are at increased risk of psychological distress ^(18)^.

For nursing professionals, the demands of professional and social life, together with exposure to occupational risks, stress and violence, lead to physical and mental fatigue, as well as burnout, increased turnover in sectors and the intention to leave the workplace. Furthermore, even when they experience stress and violence, doctors and nurses remain in the workplace because they are afraid of the repercussions for the institution, or even of being seen as weak by their colleagues ^(19 - 20)^. Likewise, many psychosocial characteristics of the work environment can influence professional burnout ^(21)^.

It is known that violence in the workplace is seen as a complex situation, in which the aggressors tend to be individuals from outside the labor institution, as well as co-workers, who can perpetuate various types of aggression, whether they are mild aggressions or aggressions that cause severe damage to the victim. Violence at work against nursing professionals leads to psychological and moral problems and physical injuries, causing problems for the labor institution and the patients cared for by these professionals ^(22)^.

When asked if they had suffered violence at work, 44.0% said they had, as did participants in a study of nursing workers in urgent and emergency care units in São Paulo, in which 61.6% of the professionals said they had suffered violence in the last 12 months ^(23)^. This corroborates a study carried out in Italy, in which 36% of the participants said they had suffered some episode of violence in the last year ^(14)^.

In addition, a study carried out in primary, secondary and tertiary care services in Bangladesh, with different health professionals, showed a prevalence of 43.3% of violent incidents in the workplace, of which 84.6% were non-physical and 15.4% physical. Furthermore, doctors reported physical violence in the workplace, while nurses reported experiencing non-physical violence ^(24)^.

In the same vein, a study carried out in Norway with health professionals working in emergency care units showed that 79.1% of cases of violence occurred among nursing professionals, demonstrating that the class with the most contact with users was the one that suffered the most violence ^(25)^.

When asked about physical violence in the workplace, 11.9% of the professionals taking part in this study said they had suffered this type of violence in the last 12 months, and one episode without the use of weapons was reported during this period, which is similar to a study carried out by the University of Turin in hospitals in northern Italy, in which 6.8% of the participants reported physical aggression in the workplace ^(14)^.

A systematic review carried out with health professionals in Pakistan reported a growing increase in violence in the workplace, where physical violence was reported with a range from 0.7% to 67% of occurrences. In addition, this type of violence was found to be particularly prevalent among males, affecting cleaners, doctors, nurses and paramedics ^(26)^. The difference in the percentage of cases may be associated with the size of the sample and the location of the studies.

When asked about the perpetrator of violence, the professionals cited patients and family members, as in a study carried out in hospitals in northern and southern Italy, in which the different types of violence were associated with patients and companions, with nurses (i.e. female professionals) and those over 55 being the biggest victims of aggression ^(27)^.

Episodes of physical aggression affect nursing professionals and are mostly perpetuated by male aggressors, influenced by overcrowding in units, failure to communicate with users, delays in care, lack of the necessary supplies for care and impatience on the part of clients, which contributes to damage to the health of the worker, who most of the time tries to minimize the occurrences of dissatisfaction with care ^(27 - 28)^.

When asked about the feelings and damage triggered by the physical aggression suffered, the participants in the survey mainly reported exhaustion, low self-esteem, disappointment, irritation and sadness, data which corroborates a systematic review carried out in hospital units, in which the participants reported feelings of emptiness, depersonalization and low perception of their work ^(28)^. The damage caused by episodes of aggression is reinforced by a study carried out in Portugal with nursing professionals working in the public system, who reported low job satisfaction, anxiety, social dysfunction and depression as a result of violence ^(29)^.

It should be noted that the underreporting of cases of physical violence is harmful to workers and the institution, since it is not possible to have a real understanding of the events and how damaging they are for the worker and the institution ^(30)^. In addition, it should be noted that the nursing team is mostly made up of women, who culturally already suffer violence due to the authoritarianism and machismo rooted in society.

In addition, a study has shown that patients tend to accumulate anger, frustration, pain and impotence and direct them towards nursing workers; therefore, the professional who helps the wounded and sick is precisely the one who runs the greatest risk of being forgotten and violated ^(31)^.

Verbal violence is conceptualized as the act of ridiculing, humiliating, verbal intimidation and lack of respect, and psychological violence is the most prevalent among nursing staff ^(32)^. This study found that 47.7% of nursing professionals reported having suffered verbal abuse in the workplace in the last 12 months. These figures are higher than those of a Korean study carried out to assess the working conditions of nurses, which found that 11.1% of professionals had experienced this type of violence. In addition, 8% reported verbal abuse and 1.7% mentioned sexual harassment and threats ^(33)^.

In this sense, another study of 410 hospital nurses in Penang, Malaysia, found that 43.9% of the participants reported experiencing violent episodes, with 82.2% citing verbal abuse ^(34)^.

This study showed that the main perpetrators of verbal violence were patients and their families, bosses and superiors, and coworkers; similarly, a study carried out in Malaysia showed that the main perpetrators of verbal aggression were patients and their families ^(32)^. Furthermore, it was noted that females were the main perpetrators of this type of aggression, which may be associated with the fact that the nursing profession is made up of a greater number of women and that patients and their families express their displeasure and indignation verbally.

In addition, a study carried out in Korea shows that suicide is the main consequence of verbal violence among professional nurses, with co-workers being characterized as the main aggressors, perpetuating a cycle of violence in which newly hired employees suffer this type of aggression and, as they gain experience, go from being victims to perpetrators of violence, with stress and overload being the main causes cited for the cycle of violence at work ^(35)^.

As consequences of verbal violence, the participants in the survey listed feelings of sadness, disappointment, anxiety, stress, irritation and loss of job satisfaction, as well as a study that shows an increase in depression, sadness, emotional exhaustion, decreased job satisfaction and a greater possibility of errors and adverse events.

It should be noted that verbal aggression is the type of violence that causes psychological changes in nursing professionals, as this act is a form of humiliation, ridicule and intimidation for the professional ^(34)^. Similarly, a study carried out with nurses at a university hospital in Rwanda found that 55.4% of the participants had been verbally abused and 15.4% had been intimidated/harassed, and the feelings experienced by 43.6% of the professionals were to avoid thinking or talking about it, for 35.9% the constant repetition of the situation in thought and to remain constantly vigilant was the feeling experienced by 11.8% ^(37)^.

It is important to emphasize that cases of violence against co-workers have a direct impact on the nursing team and the care provided, and it is essential to provide support to the victim and report cases of witnessed violence; however, it is noted that most cases are concealed by the victim and also by the witness, for fear of reprisals from the institution and the colleagues themselves, preventing the application of appropriate measures against the aggressor ^(34 , 38)^.

Sexual harassment perpetrated against nursing staff is considered a type of serious violence, characterized as undesirable sexual acts, which can be gestures, looks, speeches, expressive behaviour of a sexual nature, in which the victim who feels intimidated can be male or female ^(39)^. When asked if they had suffered sexual harassment in the workplace, only 2.8% of the participants said they had, and the victims were female. This corroborates a study carried out in a reference hospital in Rwanda, in which only 4% of the nurses mentioned this type of violence ^(37)^. Similarly, a study carried out in Malaysia found that 0.6% of participants, especially women, reported sexual violence ^(34)^.

It is understood that when asked about sexual harassment, survey participants don’t feel comfortable reporting such events and often don’t have enough evidence to report them, feeling humiliated and ashamed of what happened, which ends up contributing to underreporting and resulting in low percentages of events, when in reality these figures can be significant.

In this study, the main perpetrators of sexual harassment were male bosses/superiors and patients, as reported by a study carried out in a mobile pre-hospital care service in the state of Rio de Janeiro, in which the main aggressors were bosses and superiors, followed by patients and coworkers, with males prevailing as the perpetrators of the violence ^(40)^. A survey of nurses at university hospitals in Egypt showed that the main aggressors were patients’ relatives and coworkers ^(39)^.

It should be noted that male bosses and superiors are reported as the subjects of sexual harassment in national and international studies, which may be associated with a feeling of intimacy with the victim, and that acts of sexual harassment can be understood as jokes, which mostly leave the victim embarrassed and intimidated. It should be noted that nursing professionals are the main caregivers for victims of sexual violence in society and, together with sexual harassment suffered in the workplace, this can directly contribute to changes in psychological health ^(40)^.

With regard to the shift in which sexual harassment occurs, nighttime was the most common, which is similar to the findings of an integrative literature review that looked at the characteristics and outcomes of sexual harassment experienced by nurses in various locations ^(41)^. Occurrences of sexual harassment at night may be associated with a reduction in the demand for services and the number of people circulating in the area, leaving the aggressors more confident to provoke such acts and the victim vulnerable to the situation.

The majority of cases of sexual harassment suffered by nursing professionals are underreported due to feelings of shame and embarrassment, as well as not knowing who to turn to in the event of an incident, and victims believing that there will be no sanction for the aggressor, leaving them discredited and resorting to talking to the aggressor so that the acts don’t continue ^(41)^.

The main causes of sexual harassment are a lack of respect and ethics towards professionals and co-workers, as well as a lack of safety and monitoring of work environments, a failure in nursing staffing, and impunity for reported cases, leaving aggressors less concerned about the consequences of sexual harassment. This violence causes physical and psychological damage to the victims, and it is essential to provide a psychological support network and treatment for the effects of the violence ^(38)^.

When the association was made between exposure to violence and the profile variables, it was observed that professionals who reported working overtime were more exposed and had a higher prevalence of general violence, verbal violence and sexual abuse, as have studies in different work environments ^(34 , 36 , 38 , 41)^. In addition, higher frequencies of general violence were observed among men and physical violence among women, which may be associated with the way individuals express themselves, with males being the main perpetrators of the various types of violence.

Furthermore, 50% of nurses reported suffering violence at work, which is associated with the fact that nurses are the professionals who manage the nursing team and coordinate care, which involves resolving cases of dissatisfaction with the care provided. This makes it easier for nurses to be exposed to violence perpetuated by their colleagues, patients and their families.

The contributions of this study cover the participating professionals and the nursing category, labor institutions, as well as users of health services, promoting a holistic view of the incidence of violence in the workplace, enabling the creation of measures to reduce the occurrence of events, to implement protocols and continuing education for professionals to know how to react to violence, as well as encouraging them to record and report aggressors. In addition, they may stimulate the development of future research on the subject and the creation of public policies aimed at workers’ health.

It should be noted that, despite the different socio-cultural realities shown in the studies, violence in the workplace is highlighted by different authors as a global problem in health services, especially in hospitals, affecting different professionals and associated with various factors.

With regard to the limitations of this research, we would highlight the cross-sectional study, which prevents the data from being generalized, as well as the fact that it did not investigate individuals who were away from work. Also, the fact that the instruments used were self-administered and made available to professionals via the media (WhatsApp, e-mail), which could be influenced by the interest of the professionals.

The study’s contributions to scientific advancement include the possibility of creating future public policies for the protection of nursing professionals, with the potential to improve the work environment, directly impacting on the care provided and labor institutions.

## Conclusion

In view of the above, it was possible to see a significant occurrence of episodes of violence at work in the last 12 months, with verbal violence being the most frequently reported. It was found that nursing professionals are prone to violence from users of health services, due to their continuous contact, and also from their own work colleagues and bosses/superiors, albeit less frequently.

Workplace violence is a frequent occurrence among nursing staff, which demonstrates the importance of public policies to improve safety at work, as well as training and continuing education for nursing workers so that they know how to react to a variety of abuses.

It also emphasizes the need to improve the physical structure of services, provide the necessary equipment and adequate human resources, as well as the importance of support from health leaders and management, so that professionals feel safe to report acts of violence. Furthermore, it is important to demystify the issue of workers’ health among nursing professionals, so that underreporting becomes less and less frequent.

In addition, it is suggested that new research into violence at work be encouraged so that it is possible to encourage reflection on reducing the number of cases and changing attitudes towards the phenomenon.

## References

[1] Känel R, Princip M, Holzgang SA, Garefa C, Rossi A, DBenz C (2023). Coronary microvascular function in male physicians with burnout and job stress: an observational study. BMC Med.

[2] Cavalheiri J. C., Pascotto C. R., Tonini N. S., Vieira A. P., Ferreto L. E., Follador F. A. (2021). Sleep quality and common mental disorder in the hospital Nursing team. Rev. Latino-Am Enfermagem.

[3] Shoman Y., El May E., Marca S. C., Wild P., Bianchi R., Bugge M. D. (2021). Predictors of Occupational Burnout: A Systematic Review. Int J Environ Res Public Health.

[4] Santos D. G., Santos E. K. A., Backes M. T. S., Giacomozzi A. I., Gomes I. E. M., Kalivala K. M. M. (2021). Nursing care for women in situations of sexual violence: integrative review. Rev Enferm UERJ.

[5] Lim Z. Y., Idris D. R., Abdullah H. M. A. L., Omar H. R. (2023). Violence toward staff in the inpatient psychiatric setting: Nurses’ perspectives: A qualitative study. Arch Psychiatr Nurs.

[6] Spelten E., Thomas B., O’Meara P., Vuuren J., McGillion A. (2020). Violence against Emergency Department nurses; Can we identify the perpetrators?. PloS One.

[7] Park J. E., Song M. R. (2022). Effects of Emergency Nurses’ Experiences of Violence, Resilience, and Nursing Work Environment on Turnover Intention: A Cross-Sectional Survey. J Emerg Nurs.

[8] Fundação Oswaldo Cruz; Conselho Federal de Enfermagem. (2017). Perfil da Enfermagem no Brasil – Relatório final [Internet].

[9] Pai D. D., Sturbelle I. C. S., Santos C., Tavares J. P., Lautert L. (2018). Physical and psychological violence perpetrated in healthcare work. Texto Contexto Enferm.

[10] Instituto Brasileiro de Geografia e Estatística. (2025). Cidades e Estados: Francisco Beltrão [Homepage].

[11] Sullivan K. M., Dean A. G. OpenEpi Epidemiologic Statistics [Homepage]. http://www.openepi.com/SampleSize/SSPropor.htm.

[12] Simões M. R. L., Barroso H. H., de-Azevedo D. S. S., Duarte A. C. M., Barbosa R. E. C., Fonseca G. C. (2020). Workplace violence among municipal health care workers in Diamantina, Minas Gerais, Brazil, 2017. Rev Bras Med Trab.

[13] Silva L. O., Silva K. T. S., Costa B. T., GFGBE Ribeiro, Casimiro F. C. C., Almeida B. C. R. (2021). Violence suffered by nursing professionals in the workplace. Rev Eletr Acervo Saude.

[14] Converso D., Sottimano I., Balducci C. (2021). Violence exposure and burnout in healthcare sector: mediating role of work ability. Med Lav.

[15] Bordignon M., Monteiro M. I. (2015). Apparent validity of a questionnaire to assess violence at work. Acta Paul Enferm.

[16] Corp I. B. M. (2023). Software IBM SPSS [Internet].

[17] Ren Z., Zhao H., Zhang X., Li X., Shi H., He M. (2022). Associations of job satisfaction and burnout with psychological distress among Chinese nurses. Curr Psychol.

[18] Volonnino G., Spadazzi F., Paola L., Ancargeli M., Pascale N., Frati P. (2024). Healthcare Workers: Heroes or Victims? Context of the Western World and Proposals to Prevent Violence. Healthcare.

[19] Cao Y., Gao L., Fan L., Jiao M., Li Y., Ma Y. (2022). The Influence of Emotional Intelligence on Job Burnout of Healthcare Workers and Mediating Role of Workplace Violence: A Cross Sectional Study. Front Public Health.

[20] Sabbar D. K., Kassim W. J. (2022). Relationship between Workplace Related Violence and Job Satisfaction among Nurses Staff. Pak J Med Health Sci.

[21] Wu Y., Wang J., Liu J., Zheng J., Liu K., Baggs J. G. (2020). The impact of work environment on workplace violence, burnout and work attitudes for hospital nurses: A structural equation modelling analysis. J Nurs Manag.

[22] Queiroz A. A., Barreto F. A. (2021). Violence in nursing work in hospital services: theoretical considerations. Rev Enferm UFPE On Line.

[23] Bordignon M., Monteiro M. I. (2021). Analysis of workplace violence against nursing professionals and possibilities for prevention. Rev Gaucha Enferm.

[24] Shahjalal M. D., Gow J., Alan M. M., Ahmed T., Chakma S. K., Mohsin F. M. (2021). Workplace Violence Among Health Care Professionals in Public and Private Health Facilities in Bangladesh. Int J Public Health.

[25] Johnsen G. E., Morken T., Baste V., Rypdal K., Palmstierna T., Johansen I. H. (2020). Characteristics of aggressive incidents in emergency primary health care described by the Staff Observation Aggression Scale – Revised Emergency (SOAS-RE). BMC Health Serv Res.

[26] Rehan S. T., Shan M., Shuja S. H., Khan Z., Hussain H. U., Ochani R. K. (2023). Workplace violence against healthcare workers in Pakistan; call for action, if not now, then when? A systematic review. Glob Health Action.

[27] La Torre G, Firenze A, Gioia LP, Perric G, Soncin M, Cremonesi D (2022). Workplace violence among healthcare workers, a multicenter study in Italy. Public Health.

[28] Vidal-Alves M. J., Pina D., Ruiz-Hernández J. A., Puente-López E., Paniagua D., Martínez-Jarreta B. (2022). (Un)Broken: Lateral violence among hospital nurses, user violence, burnout, and general health: A structural equation modeling analysis. Front Med (Lausanne).

[29] Pina D., Vidal-Alves M., Puente-López E., Maldonado-Luna A., Ruiz Cabello A. L., Magalhães T. (2022). Profiles of lateral violence in nursing personnel of the Spanish public health system. PLoS One.

[30] Berger S., Grzonka P., Frei A. I., Hunziker S., Baumann S. M., Amacher A. S. (2024). Violence against healthcare professionals in intensive care units: a systematic review and meta-analysis of frequency, risk factors, interventions, and preventive measures. Crit Care.

[31] Kafle S., Paudel S., Thapaliya A., Acharya R. (2022). Workplace violence against nurses: a narrative review. J Clin Transl Res [Internet].

[32] Amorim M. C., Sillero L. S., Pires A. S., Gomes H. F., Paula G. S., Sampaio C. E. P. (2021). Violence at work from the perspective of nursing professionals. Rev Enferm Atual In Derme [Internet].

[33] Lee J., Lee B. (2022). Psychological Workplace Violence and Health Outcomes in South Korean Nurses. Workplace Health Saf.

[34] Halim I., Syukur A. Z., David C. C. H., Hanis A., Baharudin M. H., Dzualkamal D. (2022). Workplace violence among nurses in a Penang hospital: Prevalence and risk factors. Med J Malaysia [Internet].

[35] Park S. H., Choi E. H. (2022). The Cycle of Verbal Violence Among Nurse Colleagues in South Korea. J Interpers Violence.

[36] Kim S., Lynn M. R., Baernholdt M., Kitzmiller R., Jones C. B. (2023). How Does Workplace Violence-Reporting Culture Affect Workplace Violence, Nurse Burnout, and Patient Safety?. J Nurs Care Qual.

[37] Musengamana V., Adejumo O., Bananwana G., Mukagendazena M. J., Twahirda T. S., Munyaneza E. (2022). Workplace violence experience among nurses at a selected university teaching hospital in Rwanda. Pan Afr Med J.

[38] Martins B. S., Pereira M. C. (2021). Violência ocupacional na Enfermagem. Res Soc Dev.

[39] Abo Ali EA, Saied SM, Elsabagh HM, Zayed HA (2015). Sexual harassment against nursing staff in Tanta University Hospitals. Egypt. J Egypt Public Health Assoc.

[40] Sé A. C. S., Machado W. C. A., Silva P. S., Passos J. P., Araújo S. T. C., Tonini T. (2020). Physical violence, verbal abuse and sexual harassment suffered by pre-hospital care nurses. Enferm Foco.

[41] Sousa R. N., Bomfim V. V. B. S., Lins A. M. P. S., Bomfim C. V. B. S., Silva A. F., Silva M. E. W. B. (2021). Sexual harassment suffered by nursing professionals in health institutions. Res Soc Dev.

