# Supplementary material for: Violence at work experienced by nursing professionals working in hospital units: an exploratory and correlational study
Source: Rev Lat Am Enfermagem. 2025 Mar 31;33:e4527. doi: 10.1590/1518-8345.7451.4527 (PMC11960617; doi:10.1590/1518-8345.7451.4527)
Supplement: Translation

# Translation: Violência no trabalho vivenciada por profissionais de enfermagem atuantes em unidades hospitalares: uma pesquisa exploratória e correlacional*

**DOI:** 10.1590/1518-8345.7451.4528

**Authors:** Eduarda dos Santos Amaral, Gisele Arruda, Alessandro Rodrigues Perondi, Jolana Cristina Cavalheiri, Ana Paula Vieira, Franciele Ani Caovilla Follador

**Affiliations:** 1 Universidade Estadual do Oeste do Paraná, Centro de Ciências da Saúde, Francisco Beltrão, PR, Brasil; 2 Bolsista da Coordenação de Aperfeiçoamento de Pessoal de Nível Superior (CAPES), Brasil; 3 Universidade Paranaense, Curso de Enfermagem, Francisco Beltrão, PR, Brasil

**Keywords:** Violência no Trabalho, Enfermagem, Exposição à Violência, Comportamento Social, Condições de Trabalho, Saúde Ocupacional

## Abstract

identificar a ocorrência de violência no trabalho que acomete profissionais de enfermagem atuantes na área hospitalar e relacionar as variáveis de perfil profissional com esse fenômeno.

pesquisa exploratória, transversal, descritiva, correlacional, de campo e quantitativa realizada com 218 profissionais de enfermagem atuantes em unidades hospitalares da 8° Regional de Saúde do Paraná, sendo utilizado um questionário sociodemográfico e o instrumento Questionário de Avaliação da Violência no Trabalho Sofrida ou Testemunhada por Trabalhadores de Enfermagem. A análise dos dados ocorreu por frequência absoluta e relativa, utilizado o teste de Qui-quadrado com correção de continuidade de Yates para verificar os fatores associados.

participaram da amostra 218 profissionais de enfermagem, no qual 44,0% referiram ter sofrido violência no trabalho, 11,9% a violência física, 47,7% o abuso verbal e 2,8% o assédio sexual. Ao realizar-se a associação, observou-se que os profissionais com mais de 30 anos e que fazem hora extra sofrem mais violência quando comparados aos demais profissionais.

diante do exposto, foi possível evidenciar significativa ocorrência de episódios de violência no trabalho nos últimos 12 meses, sendo a violência verbal a mais referida.

## Destaques

**(1)** Prevalência significativa de violência no ambiente laboral. **(2)** Ênfase na saúde do trabalhador voltada a profissionais de enfermagem. **(3)** O abuso emocional foi o trauma na infância com maior influência na DPP. **(4)** Necessidade de políticas públicas para aprimoramento na segurança do trabalho. **(5)** Estímulo a novas pesquisas sobre a temática.

## Introdução

O trabalho está presente na sociedade de forma assídua, sendo imprescindível para o desenvolvimento pessoal e profissional, assim como para o progresso econômico e social ^(1)^. Todavia, com o avanço de novas tecnologias, as atividades laborais têm exigido sempre mais conhecimento e comprometimento dos profissionais, o que desencadeia sobrecarga e desgaste dos indivíduos ^(2)^.

Ademais, o estresse e a agitação do dia-a-dia podem acarretar diversas comorbidades aos profissionais, que interferem diretamente na qualidade de vida da população ^(3)^. Dentre os fatores desencadeantes de doenças psicossomáticas, a violência no ambiente de trabalho destaca-se como uma das principais causas de adoecimento associada à laboração ^(4)^.

Os trabalhadores da área da saúde vivenciam a violência no ambiente de trabalho em todos os seus aspectos (geral, física, verbal e sexual), sobretudo os da enfermagem, por estarem em contato direto com os pacientes e seus familiares por um tempo prolongado, ser a linha de frente da assistência em saúde, estar presente nos momentos de dor, angústia, sofrimento e morte, tornando a equipe suscetível à violência ^(5)^.

A ocorrência de violência pode desencadear danos à instituição e à saúde do trabalhador, sendo que as consequências para o âmbito institucional constituem-se no absenteísmo e presenteísmo, diminuição do comprometimento organizacional e redução da qualidade do trabalho prestado. Já para o trabalhador pode instigar repercussões psicológicas como Síndrome de *Burnout* e transtornos psíquicos menores, lesões físicas, sociais, violação da integridade pessoal, de seus direitos e dignidade ^(6)^.

Além disso, vivenciar a exposição repetida à violência leva ao desenvolvimento de respostas fisiológicas e psicológicas, como depressão, ansiedade, distúrbio do sono e isolamento, incitando muitas vezes à necessidade do uso de medicamentos, o que pode diminuir a produtividade e qualidade do atendimento prestado ^(7)^.

Um estudo realizado pelo Conselho Regional de Enfermagem de São Paulo ^(8)^ entre 2015 e 2017, com 8.332 correspondentes, demonstrou que 74% dos profissionais sofreram violência no ambiente de trabalho, sendo que 73% afirmaram que os incidentes continuaram acontecendo. Já uma pesquisa realizada na Região Sul do Brasil apontou que 63% dos participantes foram vítimas de violência nos últimos 12 meses, sendo a psicológica a mais referida (48,7%) ^(9)^.

Dessa forma, o desenvolvimento deste estudo contribuirá para uma avaliação da violência sofrida pelos profissionais atuantes em unidades hospitalares, propiciando reconhecer as lacunas que dificultam a diminuição desses atos e instituir ações que possam auxiliar na redução dos casos. Sendo assim, possibilitará o desenvolvimento de políticas públicas voltadas à área da saúde do trabalhador, visto que na região Sudoeste do Paraná não há estudos suficientes sobre o referido assunto. Ainda, os estudos sobre violência institucional podem contribuir para o desenvolvimento de pesquisas futuras, possibilitar a reflexão crítica sobre o fenômeno da violência na sociedade e como ela repercute sobre os serviços de saúde, bem como oportunizar a fundamentação de legislações trabalhistas específicas para os profissionais de saúde.

Partindo dessa premissa, o objetivo do presente estudo foi identificar a ocorrência de violência no trabalho que acomete profissionais de enfermagem atuantes na área hospitalar e relacionar as variáveis de perfil profissional com esse fenômeno.

## Método

### Tipo de estudo

Trata-se de uma pesquisa exploratória, transversal, descritiva, correlacional e quantitativa realizada com profissionais de enfermagem atuantes em unidades hospitalares.

### Ambiente do estudo

A pesquisa foi realizada no estado do Paraná, Brasil, que possui uma população de 11.443.208 habitantes, segundo dados do último censo realizado no ano de 2022 ^(10)^. A região Sudoeste, onde foi desenvolvido o estudo, abrange 27 municípios paranaenses, subdividida em 13 hospitais de pequeno, médio e grande porte que fornecem serviços de menor complexidade ou urgência e emergência, em diferentes especialidades, todas via Sistema Único de Saúde - SUS.

### Participantes do estudo

A população do estudo foi composta por todos os profissionais de enfermagem atuantes nas unidades hospitalares com atendimento via SUS da 8° Regional de Saúde do Paraná, que foram convidados a participar da pesquisa, contabilizando 744 trabalhadores. Desse total, participaram do estudo 218 profissionais de enfermagem, 91 enfermeiros (41,7%) e 127 técnicos de enfermagem (58,3%).

O tamanho da amostra foi calculado segundo a aplicação *Open Source Epidemiologic Statistics for Public Health* (2022) ^(11)^, que indicou a necessidade de, no mínimo, 209 participantes de modo a atingir um poder de 80% e um nível de significância igual ou menor a 0,05. Para este cálculo, utilizou-se como base os dados da literatura recente do Brasil, segundo a qual a prevalência da violência no trabalho é de 40% ^(8 - 14)^. Assim, estimou-se no cálculo uma população (N) de 744 indivíduos (563 técnicos; 181 enfermeiros) em 11 municípios, totalizando 13 unidades hospitalares para a coleta de dados. Logo, através de prevalência média de 40% de (±5%) em pessoas (p) em um modelo em que os limites do intervalo de confiança sejam de 95% (d) com um efeito do delineamento (ED) de 1 (amostra randômica).

### Critérios de inclusão e exclusão

Foram convidados para participar da pesquisa os profissionais de enfermagem que estavam atuando há pelo menos 30 dias na instituição, sendo excluídos os que não estavam atuando na unidade no tempo estipulado pelos pesquisadores, além dos profissionais que se encontravamem período de férias.

### Instrumento de coleta de dados

A coleta de dados foi realizada entre os meses de fevereiro a agosto de 2023, por meio de questionários autorrespondidos, via formulário *online*, que teve por objetivo identificar o perfil dos profissionais, sendo confeccionado pelos pesquisadores mediante dados da literatura, investigando as variáveis de idade, sexo, escolaridade, cor da pele, situação conjugal, categoria profissional, tempo de trabalho na instituição, cargo de chefia, se possui mais de um vínculo empregatício, turno de trabalho e se já sofreu violência no trabalho.

O segundo instrumento foi um questionário validado no Brasil, baseado no modelo da Organização Mundial da Saúde, Organização Internacional do Trabalho e de Serviços Públicos e Conselho Internacional de Enfermagem, intitulado Questionário de Avaliação da Violência no Trabalho Sofrida ou Testemunhada por Trabalhadores de Enfermagem ^(15)^. O questionário é composto por 54 questões, distribuídas em violência física no ambiente de trabalho (17 questões), abuso verbal no ambiente de trabalho (16 questões), assédio sexual no ambiente de trabalho (16 questões), outros tipos de violência no ambiente de trabalho referidos pelo trabalhador (3 questões), prevenção e redução da violência no ambiente de trabalho (3 questões), abordando os episódios sofridos, gênero do agressor, formas de prevenção, etc ^(15)^.

### Análise de dados

Antes das análises, foram realizadas a verificação e recodificação de variáveis. Para a descrição das informações, utilizaram-se as frequências absoluta e relativa. Para verificar os fatores associados aos diferentes tipos de violência no trabalho, foi utilizado o teste de Qui-quadrado com correção de continuidade de Yates. As análises foram realizadas no programa SPSS 29.0 ^(16)^, assumindo um nível de significância de p <0,05.

### Procedimentos éticos

Por ser uma pesquisa que envolve seres humanos, o estudo foi submetido primeiramente ao Comitê de Ética em Pesquisa, assim como solicitou-se autorização com o consentimento informado das instituições escolhidas como ambiente de estudo, antes da coleta de dados. Após o consentimento de ambos, realizou-se a coleta de dados, com a amostra selecionada, mediante aceitação do Termo de Consentimento Livre e Esclarecido (TCLE). Os dados foram coletados por meio de formulário online enviado para os participantes via meios eletrônicos (como *WhatsApp*, *e-mail*, por exemplo).

Foram preservados os aspectos éticos e legais indispensáveis para a pesquisa científica, além do sigilo e confidencialidade dos dados dos participantes, respeitando a Resolução 466/2012 do Conselho Nacional de Saúde. O projeto foi encaminhado ao Comitê de Ética, sendo aprovado mediante número do parecer 5.623.032.

## Resultados

Na Tabela 1 são apresentadas as principais características da amostra. Observa-se que a maioria dos participantes era do sexo feminino, com mais de 30 anos, de cor da pele branca, técnicos de enfermagem, sem cargos de chefia. Com relação aos tipos de violência, a violência verbal teve predomínio (47,7%), enquanto a violência física (11,9%) e o abuso sexual (2,8%) foram menos reportados. Aproximadamente 11,0% da amostra referiu ter doenças crônicas.

**Tabela 1 -:** Caracterização da amostra (n = 218). Sudoeste do Paraná, PR, Brasil, 2023

	**n**	**%**
**Sexo**		
Feminino	177	81,2
Masculino	41	18,8
**Escolaridade**		
Ensino Técnico completo	109	50,0
Ensino Superior incompleto	19	8,7
Ensino Superior completo	90	41,3
**Idade**		
20 a 30 anos	75	34,4
Mais de 30 anos	143	65,6
**Situação conjugal**		
Solteiro	90	41,3
Casado	116	53,2
Divorciado/Viúvo	12	5,5
**Cor**		
Branca	162	74,3
Parda	49	22,5
Negra	7	3,2
**Categoria**		
Enfermeiro	91	41,7
Técnico de Enfermagem	127	58,3
**Faz hora extra?**		
Sim	86	39,4
Não	132	60.6
**Possui cargo de chefia?**		
Sim	28	12,8
Não	190	87,2
**Turno de trabalho**		
Diurno	119	54,6
Noturno	69	31,7
Ambos	30	13,8
**Sofreu violência no ambiente de trabalho?**		
Sim	96	44,0
Não	122	56,0
**Sofreu violência física no ambiente de trabalho?**		
Sim	26	11,9
Não	192	88,1
**Sofreu violência verbal no ambiente de trabalho?**		
Sim	104	47,7
Não	114	52,3
**Sofreu assédio sexual no ambiente de trabalho?**		
Sim	6	2,8
Não	210	97,2

Os fatores associados aos diferentes tipos de violência são apresentados na Tabela 2. Foram encontradas diferenças estatisticamente significantes (p <0,05%) com relação ao trabalho em hora extra, que indicaram que os participantes que reportaram fazer hora extra apresentaram maior predomínio de violência geral, violência verbal e abuso sexual. Observam-se diferenças descritivas, que apontaram que há maior frequência de violência geral entre os homens, porém a violência física é mais frequente entre as mulheres. Ainda, de maneira descritiva, as maiores ocorrências de violência foram observadas entre os mais velhos (verbal e geral) e entre divorciados/viúvos (geral e sexual).

**Tabela 2 -:** Fatores associados à violência em profissionais de Enfermagem. Sudoeste do Paraná, PR, Brasil, 2023

**Exposições**	**Violência geral**	**Violência física**	**Violência verbal**	**Abuso sexual**
	**%**	**%**	**%**	**%**
**Sexo**				
Feminino	40,7	13,0	45,8	2,8
Masculino	58,5	7,3	56,1	2,6
p	0,057	0,457	0,308	0,929
**Escolaridade**				
Ensino Técnico completo	40,4	14,7	45,0	4,6
Ensino Superior incompleto	36,8	0,0	36,8	0,0
Ensino Superior completo	50,0	11,1	53,3	1,1
p	0,318	0,181	0,305	0,245
**Idade**				
20 a 30 anos	41,3	8,0	48,0	1,4
Acima de 30 anos	45,5	14,0	47,6	3,5
p	0,661	0,195	0,950	0,357
**Situação conjugal**				
Solteiro	48,9	8,9	52,2	1,1
Casado	39,7	12,9	42,2	4,3
Divorciado/viúvo	50,0	25,0	66,7	0,0
p	0,380	0,240	0,146	0,318
**Cor**				
Branca	42,0	9,9	45,1	3,1
Parda	51,0	20,4	59,2	2,1
Negra	42,9	0,0	28,6	0,0
p	0,534	0,070	0,128	0,760
**Categoria**				
Enfermeiro	50,5	9,9	53,8	2,2
Técnico de Enfermagem	39,4	13,4	43,3	3,2
p	0,133	0,566	0,162	0,675
**Faz hora extra**				
Sim	**54,7**	14,0	**65,1**	**6,0**
Não	**37,1**	10,6	**36,4**	**0,8**
p	**0,016**	0,595	**<0,001**	**0,024**
**Possui cargo de chefia**				
Sim	46,4	17,9	50,0	3,7
Não	43,7	11,1	47,4	2,6
p	0,785	0,469	0,795	0,754
**Turno de trabalho**				
Diurno	47,1	12,6	51,3	2,6
Noturno	39,1	7,2	43,5	1,4
Ambos	43,3	20,0	43,3	6,7
p	0,571	0,187	0,515	0,341

Na Tabela 3 são apresentados o perfil dos agressores e as consequências reportadas por cada tipo de violência. É possível observar que os homens são os principais agressores, tanto para violência física como abuso sexual. Os pacientes foram os principais agressores para violência física, enquanto os chefes/superiores foram os principais agressores para abuso sexual. Em termos de consequências, os comprometimentos mais reportados foram ansiedade, decepção, estresse, irritação e perda de satisfação com o trabalho.

**Tabela 3 -:** Perfil dos agressores e consequências de cada tipo de violência. Sudoeste do Paraná, PR, Brasil, 2023

**Exposições**	**Violência física**	**Violência verbal**	**Abuso sexual**
	**%**	**%**	**%**
**Agressor**			
Paciente	74,1	51,0*	33,3
Familiar do paciente	25,9	46,2	0,0
Chefe/superior	0,0	33,7	66,7
**Sexo do agressor**			
Feminino	22,2	41,6	0,0
Masculino	77,8	58,4	100
Consequências*			
Afastamento do trabalho	0,0	2,0	0,0
Ansiedade	22,2	37,0	33,3
Baixa autoestima	29,6	29,0	33,3
Cansaço	18,5	22,0	0,0
Crises de choro	29,6	17,0	33,3
Decepção	59,3	46,0	33,3
Dificuldade para dormir	14,8	13,0	33,3
Dor	11,1	1,0	16,7
Estresse	33,3	70,0	100
Irritação	40,7	58,0	83,3
Lesão corporal	0,0	0,0	0,0
Medo	11,1	12,0	16,7
Perda da concentração	7,4	13,0	50,0
Perda de satisfação com o trabalho	14,8	40,0	83,3
Raiva	14,8	27,0	66,7
Sentimento de inferioridade	11,1	34,0	83,3
Tristeza	37,0	36,0	50,0

*Questões separadas, por isso somam mais de 100%

## Discussão

Os profissionais de enfermagem têm papel crucial na manutenção, aprimoramento e melhoria da conscientização de saúde das pessoas, assim como são fundamentais no atendimento hospitalar e nos demais serviços de saúde ^(17)^. Em contrapartida, devido às múltiplas demandas, alta carga de trabalho e jornada laboral por turnos, os trabalhadores da área da saúde apresentam risco aumentado de sofrimento psíquico ^(18)^.

Para os profissionais de enfermagem, as demandas da vida profissional e social, juntamente com a exposição a riscos ocupacionais, estresse e violência, levam à fadiga física e mental, bem como ao esgotamento, ao aumento da rotatividade nos setores e à intenção de deixar o ambiente de trabalho. Além disso, médicos e enfermeiros, mesmo quando vivenciam situações de estresse e violência, permanecem no ambiente de trabalho por terem medo das repercussões geradas na instituição, ou mesmo de serem vistos como fracos pelos colegas de trabalho ^(19 - 20)^. Da mesma forma, muitas características psicossociais do ambiente de trabalho podem influenciar o esgotamento profissional ^(21)^.

Sabe-se que a violência no ambiente laboral é vista como uma situação complexa, em que os agressores tendem a ser indivíduos de fora da instituição trabalhista, bem como colegas de trabalho, que podem perpetuar os diversos tipos de agressões, sendo elas agressões leves ou agressões que geram danos severos à vítima. A violência no trabalho em relação ao profissional de enfermagem acarreta agravos psíquicos, morais e lesões físicas, desencadeando problemas à instituição trabalhista e aos pacientes atendidos por esses profissionais ^(22)^.

Ao serem indagados em relação a sofrer violência no trabalho, 44,0% referiram que sim, assim como participantes de estudo realizado com trabalhadores de enfermagem atuantes em unidades de urgência e emergência de São Paulo, em que 61,6% dos profissionais referiram ter sofrido violência nos últimos 12 meses ^(23)^. Tal dado corrobora estudo realizado na Itália, em que 36% dos participantes afirmaram ter sofrido algum episódio de violência no último ano ^(14)^.

Além disso, um estudo desenvolvido em serviços de atendimento primário, secundário e terciários de Bangladesh, com diferentes profissionais de saúde, evidenciou a prevalência de 43,3% de incidentes violentos no ambiente de trabalho, sendo 84,6% não físicos e 15,4% físicos. Ademais, os médicos evidenciaram a violência física no ambiente de trabalho, enquanto os enfermeiros referiram a violência não física como experienciada ^(24)^.

No mesmo sentido, pesquisa realizada na Noruega com profissionais de saúde atuantes em unidades de atendimentos emergenciais demonstrou que 79,1% dos casos de violência ocorreram com profissionais de enfermagem, demonstrando que a classe com maior contato com os usuários foi a que mais sofreu violência ^(25)^.

Quando indagados sobre violência física no ambiente de trabalho, 11,9% dos profissionais participantes deste estudo aludiram ter sofrido esse tipo de violência nos últimos 12 meses, sendo relatado um episódio sem uso de armas durante esse período, dado que se assemelha a estudo realizado pela Universidade de Turim em hospitais do norte da Itália, no qual 6,8% dos participantes referiram agressões físicas no ambiente de trabalho ^(14)^.

Uma revisão sistemática realizada com profissionais de saúde do Paquistão informou um crescente aumento da violência no ambiente de trabalho, em que a violência física foi relatada com variação de 0,7% a 67% das ocorrências. Além disso, esse tipo de violência foi evidenciado especialmente em relação sexo masculino, atingindo profissionais auxiliares de limpeza, médicos, enfermeiros e paramédicos ^(26)^. A diferença de porcentagem dos casos pode estar associada ao tamanho da amostra e local de realização dos estudos.

Quando indagados sobre o perpetuador da violência, os profissionais citaram os pacientes e familiares, assim como em estudo desenvolvido em hospitais do norte e sul da Itália, no qual os diferentes tipos de violência foram associados aos pacientes e acompanhantes, sendo as enfermeiras (ou seja, profissionais do sexo feminino) e com mais de 55 anos as maiores vítimas de agressão ^(27)^.

Os episódios de agressões físicas acometem os profissionais de enfermagem, sendo perpetuados, em sua maioria, por agressores do sexo masculino, influenciados por superlotação das unidades, falha na comunicação com os usuários, demora dos atendimentos, falta de insumos necessários para a assistência e impaciência dos clientes, o que contribui para danos à saúde do trabalhador, que, na maioria das vezes, tenta minimizar as ocorrências de insatisfação em relação ao atendimento ^(27 - 28)^.

Quando questionados sobre os sentimentos e danos desencadeados pela agressão física sofrida, os participantes da pesquisa referiram principalmente exaustão, baixa autoestima, decepção, irritação e tristeza, dados esses que corroboram revisão sistemática realizada em unidades hospitalares, em que os participantes relataram sentimento de vazio, despersonalização e baixa percepção sobre o trabalho ^(28)^. Os danos ocasionados pelos episódios de agressão são reforçados por estudo efetuado em Portugal com profissionais de enfermagem atuantes no sistema público, os quais referiram baixa satisfação com o trabalho, ansiedade, disfunção social e depressão decorrentes da violência ^(29)^.

Salienta-se que a subnotificação dos casos de violência física é deletéria para os trabalhadores e para a instituição, visto que não é possível ter entendimento real dos acontecimentos e de quão prejudiciais são para o servidor e instituição ^(30)^. Além disso, ressalta-se que a equipe de enfermagem é composta majoritariamente por mulheres, as quais, culturalmente, já sofrem violência devido ao autoritarismo e machismo enraizados na sociedade.

Ademais, estudo demonstrou que os pacientes tendem a acumular raiva, frustrações, dores e impotência, direcionando-os aos trabalhadores de enfermagem; sendo assim, o profissional que ajuda os feridos e adoentados é justamente o que corre o maior risco de ser esquecido e violentado ^(31)^.

A violência verbal é conceituada como o ato de ridicularizar, humilhar, deflagrar intimidações verbais e falta de respeito, sendo que a violência psicológica é a predominante na equipe de enfermagem ^(32)^. Neste estudo, evidenciou-se que 47,7% dos profissionais de enfermagem referiram ter sofrido agressões verbais no ambiente de trabalho nos últimos 12 meses. São dados superiores a um estudo coreano realizado para evidenciar as condições de trabalho dos enfermeiros, o qual encontrou que 11,1% dos profissionais experienciaram esse tipo de violência. Ainda, 8% reportaram abuso verbal e 1,7% aludiram a importunação sexual e ameaças ^(33)^.

Nesse sentido, um outro estudo realizado com 410 enfermeiros hospitalares de Penang, na Malásia, obteve relatos da ocorrência de episódios violentos por parte de 43,9% dos participantes, sendo o abuso verbal citado por 82,2% ^(34)^.

Neste estudo, evidenciou-se que os principais agressores relacionados à violência verbal foram os pacientes e seus familiares, chefes e superiores e colegas de trabalho; da mesma forma, estudo realizado na Malásia demonstrou que os principais perpetuadores da agressão verbal foram pacientes e seus familiares ^(32)^. Ademais, notou-se que nesse tipo de agressão o sexo feminino foi o principal agressor, o que pode estar associado ao fato de a categoria da enfermagem ser composta em maior número por mulheres e as pacientes e familiares expressarem o desgosto e indignação de forma verbal.

Além disso, estudo realizado na Coreia demonstra que o suicídio é a principal consequência da violência verbal entre os profissionais enfermeiros, sendo que os colegas de trabalho são caracterizados como os principais agressores, perpetuando um ciclo de violência no qual funcionários recém-admitidos no ambiente de trabalho sofrem esse tipo de agressão e conforme vão ganhando experiência passam de vítimas a perpetradores da violência, sendo o estresse e a sobrecarga as principais causas citadas para o ciclo da violência laboral ^(35)^.

Como consequências decorrentes da violência verbal, os participantes da pesquisa elencaram sentimentos de tristeza, decepção, ansiedade, estresse, irritação e perda de satisfação com o trabalho, assim como estudo que evidencia o aumento da depressão, tristeza, esgotamos emocional, diminuição da satisfação com o trabalho e possibilidade maior de erros e eventos adversos aos pacientes ^(36)^.

Nota-se que a agressão verbal é o tipo de violência que acarreta alterações psíquicas nos profissionais de enfermagem, pois esse ato é uma forma de humilhação, ridicularização e de intimidação para o profissional ^(34)^. Da mesma forma, estudo desenvolvido com enfermeiros em hospital universitário de Ruanda obteve que 55,4% dos participantes foram abusados verbalmente e 15,4% intimidados/assediados, e os sentimentos experienciados por 43,6% dos profissionais foram evitar pensar ou falar sobre o assunto, para 35,9% a repetição constante da situação em pensamento e manter-se em constante vigília foi o sentimento experienciado por 11,8% ^(37)^.

É importante ressaltar que os casos de violência contra colegas de trabalho repercutem diretamente sobre a equipe de enfermagem e a assistência prestada, sendo imprescindível prestar apoio à vítima e relatar os casos de violência testemunhada; porém, nota-se que a maioria dos casos são ocultados pela vítima e também pela testemunha, por medo de represálias da instituição e dos próprios colegas, impedindo a aplicação das medidas cabíveis em relação ao agressor ^(34 , 38)^.

O assédio sexual perpetuado contra a equipe de enfermagem é considerado um tipo de violência grave, caracterizada como atos sexuais indesejáveis, podendo ser gestos, olhares, falas, comportamentos expressivos de natureza sexual, em que a vítima que sente-se intimidada pode ser homem ou mulher ^(39)^. Quando indagados sobre ter sofrido assédio sexual no ambiente de trabalho, apenas 2,8% dos participantes alegaram que sim, sendo as vítimas do sexo feminino, dado que corrobora pesquisa realizada em um hospital referência de Ruanda, em que somente 4% dos enfermeiros citaram este tipo de violência ^(37)^. Da mesma forma, estudo desenvolvido na Malásia obteve 0,6% de violência sexual entre os participantes, especialmente mulheres ^(34)^.

Entende-se que quando indagados sobre o assédio sexual os participantes da pesquisa não se sentem confortáveis para relatar tais eventos e muitas vezes não possuem provas suficientes para notificar sua ocorrência, sentem-se humilhados e envergonhados pelo ocorrido, o que acaba colaborando para a subnotificação e resultando em porcentagens baixas de acontecimentos, quando na realidade esses números podem ser expressivos.

Nesta pesquisa, os principais perpetuadores do assédio sexual foram chefes/superiores e pacientes, do sexo masculino, assim como relatou um estudo efetuado em um serviço de atendimento pré-hospitalar móvel no estado do Rio de Janeiro, em que os principais agressores foram os chefes e superiores, seguidos de pacientes e colegas de trabalho, prevalecendo o sexo masculino como autor da violência ^(40)^. Uma pesquisa realizada com enfermeiras de hospitais universitários do Egito mostrou que os principais agressores foram familiares de pacientes e colegas de trabalho ^(39)^.

Nota-se que chefes e superiores do sexo masculino são relatados como sujeitos do assédio sexual em estudos nacionais e internacionais, o que pode estar associado a um sentimento de intimidade com a vítima, além de que os atos de assédio sexual podem ser entendidos como brincadeiras, que em sua maioria deixam a vítima constrangida e intimidada. Ressalta-se que as profissionais de enfermagem são as principais acolhedoras de vítimas de violência sexual na sociedade e, juntamente com o assédio sexual sofrido no ambiente de trabalho, isso pode contribuir diretamente para alteração na saúde psicológica ^(40)^.

Em relação ao turno de ocorrência do assédio sexual, evidenciou-se o período noturno como o de maior ocorrência, dado que se assemelha aos achados de uma revisão integrativa da literatura, que abordou as características e desfechos do assédio sexual vivenciado por enfermeiros de diversas localidades ^(41)^. As ocorrências de assédio sexual à noite podem estar associadas à redução da demanda de serviços e de pessoas circulantes no local, deixando os agressores mais confiantes para provocar tais atos e a vítima vulnerável à situação.

Os casos de assédio sexual sofrido por profissionais de enfermagem são em sua maioria subnotificados devido a sentimentos de vergonha e constrangimento, além de não se ter conhecimento sobre a quem recorrer perante a ocorrência, e de as vítimas acreditarem que não haverá sanção ao agressor, deixando-as desacreditadas, recorrendo a conversar com o agressor para que os atos não continuem acontecendo ^(41)^.

O assédio sexual possui como causas principais a falta de respeito e ética com os profissionais e colegas de trabalho, bem como ausência de segurança e monitorização dos ambientes trabalhistas, falha no dimensionamento de enfermagem, impunidade para os casos relatados, deixando os agressores menos preocupados com as consequências do assédio sexual deflagrado. Essa violência acarreta danos físicos e psicológicos para as vítimas, sendo imprescindível a oferta de uma rede de apoio psicológico e tratamento dos efeitos ocasionados pela violência ^(38)^.

Ao realizar a associação entre a exposição à violência e as variáveis de perfil, observou-se que os profissionais que reportaram fazer hora extra estão mais expostos e apresentaram maiores predomínios de violência geral, violência verbal e abuso sexual, assim como estudos em diferentes ambientes de trabalho ^(34 , 36 , 38 , 41)^. Além disso, foram observadas maiores frequências de violência geral entre homens e violência física entre mulheres, o que pode estar associado à forma de se expressar dos indivíduos, sendo que o sexo masculino é o principal perpetuador dos variados tipos de violência.

Ainda, 50% dos enfermeiros referiram sofrer violência no trabalho, o que associa-se ao fato de o enfermeiro ser o profissional que realiza o gerenciamento da equipe de enfermagem e que coordena os cuidados, o que envolve resolver casos de insatisfação com a assistência prestada. Este conjunto de atribuições facilita a exposição à violência perpetuada pelos próprios colegas de trabalho, pelos pacientes e seus familiares.

As contribuições deste estudo abrangem os profissionais participantes e a categoria da enfermagem, as instituições trabalhistas, bem como os usuários dos serviços de saúde, promovendo um olhar holístico sobre a incidência da violência no ambiente de trabalho, possibilitando a criação de medidas para diminuir a ocorrência dos acontecimentos, para implantar protocolos e educação continuada para os profissionais saberem como reagir diante de uma violência, além de encorajá-los a registrar e denunciar os agressores. Além disso, podem estimular o desenvolvimento de pesquisas futuras sobre a temática e a criação de políticas públicas voltadas à saúde do trabalhador.

Salienta-se que, apesar das diferentes realidades socioculturais evidenciadas nos estudos, a violência no ambiente laboral é evidenciada pelos diferentes autores como um problema global dos serviços de saúde, especialmente nos serviços hospitalares, afetando diferentes profissionais e associados a vários fatores.

Em relação às limitações desta pesquisa, destaca-se o estudo transversal, que impede a generalização dos dados, assim como a não investigação dos indivíduos afastados do trabalho. Ainda, o fato de os instrumentos utilizados serem autorrespondidos e disponibilizados aos profissionais via meios de comunicação (*WhatsApp*, *e-mail*), podendo sofrer influência do interesse dos profissionais.

As contribuições do estudo para o avanço científico englobam a possibilidade da criação de futuras políticas públicas para a proteção dos profissionais de enfermagem, tendo potencial para aprimoramento do ambiente de trabalho, impactando diretamente na assistência prestada e nas instituições trabalhistas.

## Conclusão

Diante do exposto, foi possível evidenciar a ocorrência significativa de episódios de violência no trabalho nos últimos 12 meses, sendo a violência verbal a mais referida. Constatou-se que os profissionais de enfermagem estão propensos a sofrer violência proveniente dos usuários dos serviços de saúde, pelo seu contato contínuo, e ainda dos próprios colegas de trabalho e chefes/superiores, mesmo que em uma frequência menor.

A violência no trabalho está presente na equipe de enfermagem de forma assídua, o que demonstra a importância de políticas públicas para aprimoramento da segurança no trabalho, bem como capacitação e educação continuada dos trabalhadores de enfermagem para que saibam reagir diante da variedade de abusos.

Enfatiza-se, ainda, a necessidade de melhoria na estrutura física dos serviços, o fornecimento de equipamentos necessários e adequação de recursos humanos, além de ser de suma importância o apoio das lideranças e gestão da saúde, para que os profissionais se sintam seguros em denunciar os atos de violência. Ademais, destaca-se a importância de desmistificar o tema da saúde do trabalhador junto ao profissional de enfermagem, para que a subnotificação seja cada vez menor.

Além disso, sugere-se o estímulo a novas pesquisas sobre a violência no trabalho para que, dessa forma, seja possível incitar reflexões para redução dos casos e mudança de postura perante a ocorrência do fenômeno.

# Translation: Violencia laboral experimentada por profesionales de enfermería que trabajan en unidades hospitalarias: una investigación exploratoria y correlacional*

**DOI:** 10.1590/1518-8345.7451.4526

**Authors:** Eduarda dos Santos Amaral, Gisele Arruda, Alessandro Rodrigues Perondi, Jolana Cristina Cavalheiri, Ana Paula Vieira, Franciele Ani Caovilla Follador

**Affiliations:** 1 Universidade Estadual do Oeste do Paraná, Centro de Ciências da Saúde, Francisco Beltrão, PR, Brasil; 2 Becaria de la Coordenação de Aperfeiçoamento de Pessoal de Nível Superior (CAPES), Brasil; 3 Universidade Paranaense, Curso de Enfermagem, Francisco Beltrão, PR, Brasil

**Keywords:** Violencia Laboral, Enfermería, Exposición a la Violencia, Comportamiento Social, Condiciones de Trabajo, Salud Ocupacional

## Abstract

identificar la ocurrencia de violencia en el trabajo que afecta a profesionales de enfermería que laboran en el área hospitalaria y relacionar las variables de perfil profesional con este fenómeno.

investigación exploratoria, transversal, descriptiva, correlacional, de campo y cuantitativa realizada con 218 profesionales de enfermería que trabajan en unidades hospitalarias de la *8ª Regional de Saúde do Paraná*, utilizando un cuestionario sociodemográfico y el instrumento Cuestionario de Evaluación de la Violencia en el Trabajo Sufrida o Presenciada por Trabajadores de Enfermería. El análisis de los datos se realizó con frecuencia absoluta y relativa, utilizando la prueba de Chi-cuadrado con corrección de continuidad de Yates para verificar los factores asociados.

participaron en la muestra 218 profesionales de enfermería, de los cuales el 44,0% reportó haber sufrido violencia en el trabajo, el 11,9% violencia física, el 47,7% abuso verbal y el 2,8% acoso sexual. Al realizar la asociación, se observó que los profesionales con más de 30 años y que hacen horas extra sufren más violencia en comparación con los demás profesionales.

ante lo expuesto, fue posible evidenciar una significativa ocurrencia de episodios de violencia en el trabajo en los últimos 12 meses, siendo la violencia verbal la más referida.

## Destacados

**(1)** Prevalencia significativa de violencia en el ambiente laboral. **(2)** Énfasis en la salud del trabajador dirigida a profesionales de enfermería. **(3)** La violencia verbal afecta al 47,7% de la muestra estudiada. **(4)** Necesidad de políticas públicas para mejorar la seguridad en el trabajo. **(5)** Estímulo a nuevas investigaciones sobre la temática.

## Introducción

El trabajo está presente en la sociedad de forma asidua, siendo imprescindible para el desarrollo personal y profesional, así como para el progreso económico y social ^(1)^. Sin embargo, con el avance de nuevas tecnologías, las actividades laborales han exigido siempre más conocimiento y compromiso de los profesionales, lo que desencadena sobrecarga y desgaste de los individuos ^(2)^.

Además, el estrés y la agitación del día a día pueden acarrear diversas comorbilidades a los profesionales, que interfieren directamente en la calidad de vida de la población ^(3)^. Entre los factores desencadenantes de enfermedades psicosomáticas, la violencia en el ambiente de trabajo se destaca como una de las principales causas de enfermedad asociada al trabajo ^(4)^.

Los trabajadores del área de la salud experimentan la violencia en el ambiente laboral en todos sus aspectos (general, física, verbal y sexual), sobre todo los de enfermería, por estar en contacto directo con los pacientes y sus familiares por un tiempo prolongado, ser la línea de frente de la asistencia en salud, estar presentes en momentos de dolor, angustia, sufrimiento y muerte, haciendo al equipo susceptible a la violencia ^(5)^.

La ocurrencia de violencia puede desencadenar daños a la institución y a la salud del trabajador, siendo que las consecuencias para el ámbito institucional se constituyen en absentismo y presentismo, disminución del compromiso organizacional y reducción de la calidad del trabajo prestado. Ya para el trabajador puede provocar repercusiones psicológicas como Síndrome de *Burnout* y trastornos psíquicos menores, lesiones físicas, sociales, violación de la integridad personal, de sus derechos y dignidad ^(6)^.

Además, vivir la exposición repetida a la violencia lleva al desarrollo de respuestas fisiológicas y psicológicas, como depresión, ansiedad, trastorno del sueño y aislamiento, incitando muchas veces a la necesidad del uso de medicamentos, lo que puede disminuir la productividad y calidad de la atención prestada ^(7)^.

Un estudio realizado por el *Conselho Regional de Enfermagem de São Paulo* ^(8)^ entre 2015 y 2017, con 8.332 correspondientes, demostró que el 74% de los profesionales sufrieron violencia en el ambiente de trabajo, siendo que el 73% afirmaron que los incidentes continuaron ocurriendo. Ya una investigación realizada en la Región Sur de Brasil señaló que el 63% de los participantes fueron víctimas de violencia en los últimos 12 meses, siendo la psicológica la más referida (48,7%) ^(9)^.

De esta forma, el desarrollo de este estudio contribuirá a una evaluación de la violencia sufrida por los profesionales que actúan en unidades hospitalarias, propiciando reconocer las lagunas que dificultan la disminución de estos actos e instituir acciones que puedan auxiliar en la reducción de los casos. Así, posibilitará el desarrollo de políticas públicas orientadas al área de la salud del trabajador, dado que en la región Suroeste de Paraná no hay estudios suficientes sobre el referido asunto. Además, los estudios sobre violencia institucional pueden contribuir al desarrollo de investigaciones futuras, posibilitar la reflexión crítica sobre el fenómeno de la violencia en la sociedad y cómo repercute en los servicios de salud, así como ofrecer la fundamentación de legislaciones laborales específicas para los profesionales de salud.

Partiendo de esta premisa, el objetivo del presente estudio fue identificar la ocurrencia de violencia en el trabajo que afecta a profesionales de enfermería que actúan en el área hospitalaria y relacionar las variables de perfil profesional con este fenómeno.

## Método

### Tipo de estudio

Se trata de una investigación exploratoria, transversal, descriptiva, correlacional y cuantitativa realizada con profesionales de enfermería que actúan en unidades hospitalarias.

### Ambiente del estudio

La investigación se llevó a cabo en el estado de Paraná, Brasil, que tiene una población de 11.443.208 habitantes, según datos del último censo realizado en el año 2022 ^(10)^. La región Sudoeste, donde se desarrolló el estudio, abarca 27 municipios paranaenses, subdividida en 13 hospitales de pequeño, mediano y gran porte que brindan servicios de menor complejidad o urgencia y emergencia, en diferentes especialidades, todas vía *Sistema Único de Saúde* (SUS).

### Participantes del estudio

La población del estudio estuvo compuesta por todos los profesionales de enfermería que actúan en las unidades hospitalarias con atención vía SUS de la 8ª *Regional de Saúde do Paraná*, que fueron invitados a participar en la investigación, contabilizando 744 trabajadores. De este total, participaron en el estudio 218 profesionales de enfermería, 91 enfermeros (41,7%) y 127 técnicos de enfermería (58,3%).

El tamaño de la muestra se calculó según la aplicación *Open Source Epidemiologic Statistics for Public Health* (2022) ^(11)^, que indicó la necesidad de, como mínimo, 209 participantes para alcanzar un poder del 80% y un nivel de significancia igual o menor a 0,05. Para este cálculo, se utilizó como base los datos de la literatura reciente de Brasil, según la cual la prevalencia de la violencia en el trabajo es del 40% ^(8 - 14)^. Así, se estimó en el cálculo una población (N) de 744 individuos (563 técnicos; 181 enfermeros) en 11 municipios, totalizando 13 unidades hospitalarias para la recolección de datos. Por lo tanto, mediante una prevalencia media del 40% (±5%) en personas (p) en un modelo donde los límites del intervalo de confianza sean del 95% (d) con un efecto del diseño (ED) de 1 (muestra aleatoria).

### Criterios de inclusión y exclusión

Fueron invitados a participar en la investigación los profesionales de enfermería que estaban actuando al menos 30 días en la institución, siendo excluidos los que no estaban actuando en la unidad en el tiempo estipulado por los investigadores, además de los profesionales que se encontraban en período de vacaciones.

### Instrumento de recolección de datos

La recolección de datos se realizó entre los meses de febrero a agosto de 2023, mediante cuestionarios autoadministrados, por medio de un formulario en línea, que tuvo por objetivo identificar el perfil de los profesionales, siendo confeccionado por los investigadores mediante datos de la literatura, investigando las variables de edad, sexo, escolaridad, color de piel, situación conyugal, categoría profesional, tiempo de trabajo en la institución, cargo de jefatura, si posee más de un vínculo laboral, turno de trabajo y si ya ha sufrido violencia en el trabajo.

El segundo instrumento fue un cuestionario validado en Brasil, basado en el modelo de la Organización Mundial de la Salud, Organización Internacional del Trabajo y de Servicios Públicos y Consejo Internacional de Enfermería, titulado Cuestionario de Evaluación de la Violencia en el Trabajo Sufrida o Presenciada por Trabajadores de Enfermería ^(15)^. El cuestionario está compuesto por 54 preguntas, distribuidas en violencia física en el ambiente de trabajo (17 preguntas), abuso verbal en el ambiente de trabajo (16 preguntas), acoso sexual en el ambiente de trabajo (16 preguntas), otros tipos de violencia en el ambiente de trabajo referidos por el trabajador (3 preguntas), prevención y reducción de la violencia en el ambiente de trabajo (3 preguntas), abordando los episodios sufridos, género del agresor, formas de prevención, etc. ^(15)^.

### Análisis de datos

Antes de los análisis, se realizaron la verificación y recodificación de variables. Para la descripción de las informaciones, se utilizaron las frecuencias absoluta y relativa. Para verificar los factores asociados a los diferentes tipos de violencia en el trabajo, se utilizó la prueba de Chi-cuadrado con corrección de continuidad de Yates. Los análisis se realizaron en el programa SPSS 29.0 ^(16)^, asumiendo un nivel de significancia de p <0,05.

### Procedimientos éticos

Por ser una investigación que involucra seres humanos, el estudio fue sometido primeramente al Comité de Ética en Investigación, así como se solicitó autorización con el consentimiento informado de las instituciones elegidas como ambiente de estudio, antes de la recolección de datos. Tras el consentimiento de ambos, se realizó la recolección de datos, con la muestra seleccionada, mediante la aceptación del Consentimiento Informado (CI). Los datos fueron recolectados por medio de un formulario en línea enviado a los participantes vía medios electrónicos (como *WhatsApp*, correo electrónico, por ejemplo).

Se preservaron los aspectos éticos y legales indispensables para la investigación científica, además del sigilo y confidencialidad de los datos de los participantes, respetando la Resolución 466/2012 del *Conselho Nacional de Saúde*. El proyecto fue enviado al Comité de Ética, siendo aprobado mediante el número de dictamen 5.623.032.

## Resultados

En la Tabla 1 se presentan las principales características de la muestra. Se observa que la mayoría de los participantes eran del sexo femenino, mayores de 30 años, de piel blanca, técnicos de enfermería, sin cargos de jefatura. Con relación a los tipos de violencia, la violencia verbal tuvo predominio (47,7%), mientras que la violencia física (11,9%) y el abuso sexual (2,8%) fueron menos reportados. Aproximadamente el 11,0% de la muestra refirió tener enfermedades crónicas.

**Tabla 1 -:** Caracterización de la muestra (n = 218). Sudoeste de Paraná, PR, Brasil, 2023

	**n**	**%**
**Sexo**		
Femenino	177	81,2
Masculino	41	18,8
**Escolaridad**		
Educación Técnica completa	109	50,0
Educación Superior incompleta	19	8,7
Educación Superior completa	90	41,3
**Edad**		
20 a 30 años	75	34,4
Más de 30 años	143	65,6
**Situación conyugal**		
Soltero	90	41,3
Casado	116	53,2
Divorciado/Viudo	12	5,5
**Color**		
Blanco	162	74,3
Pardo	49	22,5
Negro	7	3,2
**Categoría**		
Enfermero	91	41,7
Técnico de Enfermería	127	58,3
**¿Hace hora extra?**		
Sí	86	39,4
No	132	60.6
¿Tiene cargo de jefatura?		
Sí	28	12,8
No	190	87,2
**Turno de trabajo**		
Diurno	119	54,6
Nocturno	69	31,7
Ambos	30	13,8
¿Sufrió violencia en el ambiente de trabajo?		
Sí	96	44,0
No	122	56,0
**¿Sufrió violencia física en el ambiente de trabajo?**		
Sí	26	11,9
No	192	88,1
**¿Sufrió violencia verbal en el ambiente de trabajo?**		
Sí	104	47,7
No	114	52,3
**¿Sufrió acoso sexual en el ambiente de trabajo?**		
Sí	6	2,8
No	210	97,2

Los factores asociados a los diferentes tipos de violencia se presentan en la Tabla 2. Se encontraron diferencias estadísticamente significativas (p <0,05%) con relación al trabajo en horas extra, que indicaron que los participantes que reportaron hacer horas extra presentaron mayor predominio de violencia general, violencia verbal y abuso sexual. Se observan diferencias descriptivas que señalaron que hay mayor frecuencia de violencia general entre los hombres, pero la violencia física es más frecuente entre las mujeres. Además, de manera descriptiva, las mayores ocurrencias de violencia se observaron entre los más mayores (verbal y general) y entre divorciados/viudos (general y sexual).

**Tabla 2 -:** Factores asociados a la violencia en profesionales de Enfermería. Sudoeste de Paraná, PR, Brasil, 2023

**Exposiciones**	**Violencia general**	**Violencia fisica**	**Violencia verbal**	**Abuso sexual**
	**%**	**%**	**%**	**%**
**Sexo**				
Femenino	40,7	13,0	45,8	2,8
Masculino	58,5	7,3	56,1	2,6
p	0,057	0,457	0,308	0,929
**Escolaridad**				
Educación Técnica completa	40,4	14,7	45,0	4,6
Educación Superior incompleta	36,8	0,0	36,8	0,0
Educación Superior completa	50,0	11,1	53,3	1,1
p	0,318	0,181	0,305	0,245
**Edad**				
20 a 30 años	41,3	8,0	48,0	1,4
Más de 30 años	45,5	14,0	47,6	3,5
p	0,661	0,195	0,950	0,357
**Situación conyugal**				
Soltero	48,9	8,9	52,2	1,1
Casado	39,7	12,9	42,2	4,3
Divorciado/viudo	50,0	25,0	66,7	0,0
p	0,380	0,240	0,146	0,318
**Color**				
Blanco	42,0	9,9	45,1	3,1
Pardo	51,0	20,4	59,2	2,1
Negro	42,9	0,0	28,6	0,0
p	0,534	0,070	0,128	0,760
**Categoría**				
Enfermero	50,5	9,9	53,8	2,2
Técnico de Enfermería	39,4	13,4	43,3	3,2
p	0,133	0,566	0,162	0,675
**Hace hora extra**				
Sí	**54,7**	14,0	**65,1**	**6,0**
No	**37,1**	10,6	**36,4**	**0,8**
p	**0,016**	0,595	**<0,001**	**0,024**
**Tiene cargo de jefe**				
Sí	46,4	17,9	50,0	3,7
No	43,7	11,1	47,4	2,6
p	0,785	0,469	0,795	0,754
**Turno de trabajo**				
Diurno	47,1	12,6	51,3	2,6
Nocturno	39,1	7,2	43,5	1,4
Ambos	43,3	20,0	43,3	6,7
p	0,571	0,187	0,515	0,341

En la Tabla 3 se presentan el perfil de los agresores y las consecuencias reportadas por cada tipo de violencia. Es posible observar que los hombres son los principales agresores, tanto para violencia física como para abuso sexual. Los pacientes fueron los principales agresores para violencia física, mientras que los jefes/superiores fueron los principales agresores para abuso sexual. En términos de consecuencias, los compromisos más reportados fueron ansiedad, decepción, estrés, irritación y pérdida de satisfacción con el trabajo.

**Tabla 3 -:** Perfil de los agresores y consecuencias de cada tipo de violencia. Sudoeste de Paraná, PR, Brasil, 2023

**Exposiciones**	**Violencia fisica**	**Violencia verbal**	**Abuso sexual**
	**%**	**%**	**%**
**Agresor**			
Paciente	74,1	51,0*	33,3
Familiar del paciente	25,9	46,2	0,0
Jefe/superior	0,0	33,7	66,7
**Sexo del agresor**			
Femenino	22,2	41,6	0,0
Masculino	77,8	58,4	100
Consecuencias*			
Ausencia laboral	0,0	2,0	0,0
Ansiedad	22,2	37,0	33,3
Baja autoestima	29,6	29,0	33,3
Cansancio	18,5	22,0	0,0
Crisis de llanto	29,6	17,0	33,3
Decepción	59,3	46,0	33,3
Dificultad para dormir	14,8	13,0	33,3
Dolor	11,1	1,0	16,7
Estrés	33,3	70,0	100
Irritación	40,7	58,0	83,3
Lesión corporal	0,0	0,0	0,0
Miedo	11,1	12,0	16,7
Pérdida de concentración	7,4	13,0	50,0
Pérdida de satisfacción con el trabajo	14,8	40,0	83,3
Rabia	14,8	27,0	66,7
Sentimiento de inferioridad	11,1	34,0	83,3
Tristeza	37,0	36,0	50,0

*Preguntas separadas, por eso suman más del 100%

## Discusión

Los profesionales de enfermería tienen un papel crucial en el mantenimiento, mejoramiento y aumento de la concientización sobre la salud de las personas, así como son fundamentales en la atención hospitalaria y en los demás servicios de salud ^(17)^. Por otro lado, debido a las múltiples demandas, alta carga de trabajo y jornada laboral por turnos, los trabajadores del área de la salud presentan un riesgo aumentado de sufrimiento psíquico ^(18)^.

Para los profesionales de enfermería, las demandas de la vida profesional y social, junto con la exposición a riesgos ocupacionales, estrés y violencia, llevan a la fatiga física y mental, así como al agotamiento, al aumento de la rotación en los sectores y a la intención de abandonar el ambiente de trabajo. Además, médicos y enfermeros, incluso cuando experimentan situaciones de estrés y violencia, permanecen en el ambiente laboral por temor a las repercusiones generadas en la institución, o incluso a ser vistos como débiles por los colegas de trabajo ^(19 - 20)^. De la misma forma, muchas características psicosociales del ambiente laboral pueden influir en el agotamiento profesional ^(21)^.

Se sabe que la violencia en el ambiente laboral es vista como una situación compleja, en la que los agresores tienden a ser individuos de fuera de la institución laboral, así como colegas de trabajo, que pueden perpetuar los diversos tipos de agresiones, ya sean agresiones leves o agresiones que generan daños severos a la víctima. La violencia en el trabajo en relación con el profesional de enfermería acarrea daños psíquicos, morales y lesiones físicas, desencadenando problemas a la institución laboral y a los pacientes atendidos por esos profesionales ^(22)^.

Al ser indagados sobre si sufrieron violencia en el trabajo, el 44,0% refirieron que sí, al igual que participantes de un estudio realizado con trabajadores de enfermería que actúan en unidades de urgencia y emergencia de São Paulo, en el que el 61,6% de los profesionales refirieron haber sufrido violencia en los últimos 12 meses ^(23)^. Este dato corrobora un estudio realizado en Italia, en el que el 36% de los participantes afirmaron haber sufrido algún episodio de violencia en el último año ^(14)^.

Además, un estudio desarrollado en servicios de atención primaria, secundaria y terciaria de Bangladesh, con diferentes profesionales de salud, evidenció una prevalencia de 43,3% de incidentes violentos en el ambiente de trabajo, siendo 84,6% no físicos y 15,4% físicos. Asimismo, los médicos evidenciaron la violencia física en el ambiente de trabajo, mientras que los enfermeros refirieron la violencia no física como experimentada ^(24)^.

En el mismo sentido, una investigación realizada en Noruega con profesionales de salud que actúan en unidades de atención de emergencias demostró que el 79,1% de los casos de violencia ocurrieron con profesionales de enfermería, mostrando que la clase con mayor contacto con los usuarios fue la que más sufrió violencia ^(25)^.

Al ser indagados sobre violencia física en el ambiente de trabajo, el 11,9% de los profesionales participantes de este estudio aludieron haber sufrido este tipo de violencia en los últimos 12 meses, siendo reportado un episodio sin uso de armas durante este período, dato que se asemeja a un estudio realizado por la Universidad de Turín en hospitales del norte de Italia, en el cual el 6,8% de los participantes refirieron agresiones físicas en el ambiente de trabajo ^(14)^.

Una revisión sistemática realizada con profesionales de salud de Pakistán informó un creciente aumento de la violencia en el ambiente de trabajo, en el que la violencia física fue reportada con una variación de 0,7% a 67% de las ocurrencias. Además, este tipo de violencia fue evidenciado especialmente en relación con el sexo masculino, afectando a profesionales auxiliares de limpieza, médicos, enfermeros y paramédicos ^(26)^. La diferencia en el porcentaje de los casos puede estar asociada al tamaño de la muestra y al lugar de realización de los estudios.

Cuando se les preguntó sobre el perpetrador de la violencia, los profesionales citaron a los pacientes y familiares, al igual que en un estudio desarrollado en hospitales del norte y sur de Italia, en el cual los diferentes tipos de violencia fueron asociados a los pacientes y acompañantes, siendo las enfermeras (es decir, profesionales de sexo femenino) y mayores de 55 años las principales víctimas de agresión ^(27)^.

Los episodios de agresiones físicas afectan a los profesionales de enfermería, siendo perpetrados, en su mayoría, por agresores de sexo masculino, influenciados por la superpoblación de las unidades, fallas en la comunicación con los usuarios, demora en los atendimientos, falta de insumos necesarios para la asistencia e impaciencia de los clientes, lo que contribuye a daños en la salud del trabajador, quien, en la mayoría de las veces, intenta minimizar las ocurrencias de insatisfacción en relación con la atención ^(27 - 28)^.

Cuando se les preguntó sobre los sentimientos y daños desencadenados por la agresión física sufrida, los participantes de la investigación refirieron principalmente agotamiento, baja autoestima, decepción, irritación y tristeza, datos que corroboran una revisión sistemática realizada en unidades hospitalarias, en la que los participantes relataron sentimiento de vacío, despersonalización y baja percepción sobre el trabajo ^(28)^. Los daños ocasionados por los episodios de agresión son reforzados por un estudio efectuado en Portugal con profesionales de enfermería que actúan en el sistema público, los cuales refirieron baja satisfacción con el trabajo, ansiedad, disfunción social y depresión derivadas de la violencia ^(29)^.

Se señala que la subnotificación de los casos de violencia física es perjudicial para los trabajadores y para la institución, ya que no es posible tener un entendimiento real de los acontecimientos y de cuán perjudiciales son para el servidor y la institución ^(30)^. Además, se destaca que el equipo de enfermería está compuesto mayoritariamente por mujeres, las cuales, culturalmente, ya sufren violencia debido al autoritarismo y machismo arraigados en la sociedad.

Además, un estudio demostró que los pacientes tienden a acumular ira, frustraciones, dolores e impotencia, dirigiéndolos hacia los trabajadores de enfermería; por lo tanto, el profesional que ayuda a los heridos y enfermos es justamente quien corre el mayor riesgo de ser olvidado y violentado ^(31)^.

La violencia verbal se conceptualiza como el acto de ridiculizar, humillar, desencadenar intimidaciones verbales y falta de respeto, siendo que la violencia psicológica es la predominante en el equipo de enfermería ^(32)^. En este estudio, se evidenció que el 47,7% de los profesionales de enfermería refirieron haber sufrido agresiones verbales en el ambiente de trabajo en los últimos 12 meses. Son datos superiores a un estudio coreano realizado para evidenciar las condiciones de trabajo de los enfermeros, el cual encontró que el 11,1% de los profesionales experimentaron este tipo de violencia. Además, el 8% reportó abuso verbal y el 1,7% aludió a importunación sexual y amenazas ^(33)^.

En este sentido, otro estudio realizado con 410 enfermeros hospitalarios de Penang, en Malasia, obtuvo relatos de la ocurrencia de episodios violentos por parte del 43,9% de los participantes, siendo el abuso verbal citado por el 82,2% ^(34)^.

En este estudio, se evidenció que los principales agresores relacionados con la violencia verbal fueron los pacientes y sus familiares, jefes, superiores y colegas de trabajo; de la misma manera, un estudio realizado en Malasia demostró que los principales perpetradores de la agresión verbal fueron pacientes y sus familiares ^(32)^. Además, se notó que en este tipo de agresión el sexo femenino fue el principal agresor, lo que puede estar asociado al hecho de que la categoría de enfermería está compuesta en mayor número por mujeres y que las pacientes y familiares expresan el disgusto e indignación de forma verbal.

Además, un estudio realizado en Corea demuestra que el suicidio es la principal consecuencia de la violencia verbal entre los profesionales enfermeros, siendo que los colegas de trabajo son caracterizados como los principales agresores, perpetuando un ciclo de violencia en el cual los empleados recién admitidos en el ambiente de trabajo sufren este tipo de agresión y, conforme van ganando experiencia, pasan de víctimas a perpetradores de la violencia, siendo el estrés y la sobrecarga las principales causas citadas para el ciclo de la violencia laboral ^(35)^.

Como consecuencias derivadas de la violencia verbal, los participantes de la investigación enumeraron sentimientos de tristeza, decepción, ansiedad, estrés, irritación y pérdida de satisfacción con el trabajo, al igual que un estudio que evidencia el aumento de la depresión, tristeza, agotamiento emocional, disminución de la satisfacción con el trabajo y mayor posibilidad de errores y eventos adversos a los pacientes ^(36)^.

Se nota que la agresión verbal es el tipo de violencia que acarrea alteraciones psíquicas en los profesionales de enfermería, pues este acto es una forma de humillación, ridiculización e intimidación para el profesional ^(34)^. De la misma forma, un estudio desarrollado con enfermeros en un hospital universitario de Ruanda obtuvo que el 55,4% de los participantes fueron abusados verbalmente y el 15,4% intimidados/acosados, y los sentimientos experimentados por el 43,6% de los profesionales fueron evitar pensar o hablar sobre el asunto; para el 35,9%, la repetición constante de la situación en pensamiento, y mantenerse en constante vigilancia fue el sentimiento experimentado por el 11,8% ^(37)^.

Es importante resaltar que los casos de violencia contra colegas de trabajo repercuten directamente sobre el equipo de enfermería y la asistencia prestada, siendo imprescindible prestar apoyo a la víctima y relatar los casos de violencia presenciada; sin embargo, se nota que la mayoría de los casos son ocultados por la víctima y también por el testigo, por miedo a represalias de la institución y de los propios colegas, impidiendo la aplicación de las medidas apropiadas con relación al agresor ^(34 , 38)^.

El acoso sexual perpetuado contra el equipo de enfermería es considerado un tipo de violencia grave, caracterizada como actos sexuales no deseados, pudiendo ser gestos, miradas, palabras, comportamientos expresivos de naturaleza sexual, en que la víctima que se siente intimidada puede ser hombre o mujer ^(39)^. Al ser indagados sobre haber sufrido acoso sexual en el ambiente de trabajo, solo el 2,8% de los participantes alegaron que sí, siendo las víctimas del sexo femenino, dato que corrobora una investigación realizada en un hospital de referencia de Ruanda, en que solo el 4% de los enfermeros citaron este tipo de violencia ^(37)^. De la misma forma, un estudio desarrollado en Malasia obtuvo 0,6% de violencia sexual entre los participantes, especialmente mujeres ^(34)^.

Se entiende que cuando son indagados sobre el acoso sexual, los participantes de la investigación no se sienten cómodos para relatar tales eventos y muchas veces no poseen pruebas suficientes para notificar su ocurrencia, se sienten humillados y avergonzados por lo ocurrido, lo que termina colaborando para la subnotificación y resultando en porcentajes bajos de acontecimientos, cuando en realidad esos números pueden ser significativos.

En esta investigación, los principales perpetradores del acoso sexual fueron jefes/superiores y pacientes, del sexo masculino, así como relató un estudio efectuado en un servicio de atención prehospitalaria móvil en el estado de Rio de Janeiro, en el que los principales agresores fueron los jefes y superiores, seguidos de pacientes y colegas de trabajo, prevaleciendo el sexo masculino como autor de la violencia ^(40)^. Una investigación realizada con enfermeras de hospitales universitarios de Egipto mostró que los principales agresores fueron familiares de pacientes y colegas de trabajo ^(39)^.

Se nota que jefes y superiores de sexo masculino son reportados como sujetos del acoso sexual en estudios nacionales e internacionales, lo que puede estar asociado a un sentimiento de intimidad con la víctima, además de que los actos de acoso sexual pueden ser entendidos como bromas que, en su mayoría, dejan a la víctima avergonzada e intimidada. Se resalta que las profesionales de enfermería son las principales acogedoras de víctimas de violencia sexual en la sociedad y, junto con el acoso sexual sufrido en el ambiente de trabajo, esto puede contribuir directamente a alteraciones en la salud psicológica ^(40)^.

Con relación al turno de ocurrencia del acoso sexual, se evidenció el período nocturno como el de mayor incidencia, dato que se asemeja a los hallazgos de una revisión integrativa de la literatura, que abordó las características y desenlaces del acoso sexual vivido por enfermeros de diversas localidades ^(41)^. Las ocurrencias de acoso sexual en la noche pueden estar asociadas a la reducción de la demanda de servicios y de personas circulantes en el lugar, dejando a los agresores más confiados para provocar tales actos y a la víctima vulnerable a la situación.

Los casos de acoso sexual sufrido por profesionales de enfermería son en su mayoría subnotificados debido a sentimientos de vergüenza y constricción, además de no tener conocimiento sobre a quién recurrir ante la ocurrencia, y de que las víctimas creen que no habrá sanción al agresor, dejándolas desalentadas, recurriendo a conversar con el agresor para que los actos no continúen ocurriendo ^(41)^.

El acoso sexual tiene como causas principales la falta de respeto y ética con los profesionales y colegas de trabajo, así como la ausencia de seguridad y monitoreo de los ambientes laborales, fallas en el dimensionamiento de enfermería, impunidad para los casos reportados, dejando a los agresores menos preocupados por las consecuencias del acoso sexual desencadenado. Esta violencia acarrea daños físicos y psicológicos para las víctimas, siendo imprescindible ofrecer una red de apoyo psicológico y tratamiento de los efectos ocasionados por la violencia ^(38)^.

Al realizar la asociación entre la exposición a la violencia y las variables de perfil, se observó que los profesionales que reportaron hacer horas extra están más expuestos y presentaron mayores predominancias de violencia general, violencia verbal y abuso sexual, al igual que estudios en diferentes ambientes de trabajo ^(34 , 36 , 38 , 41)^. Además, se observaron mayores frecuencias de violencia general entre hombres y violencia física entre mujeres, lo que puede estar asociado a la forma de expresarse de los individuos, siendo que el sexo masculino es el principal perpetrador de los variados tipos de violencia.

Además, el 50% de los enfermeros refirieron sufrir violencia en el trabajo, lo que se asocia al hecho de que el enfermero es el profesional que realiza la gestión del equipo de enfermería y coordina los cuidados, lo que implica resolver casos de insatisfacción con la asistencia prestada. Este conjunto de atribuciones facilita la exposición a la violencia perpetrada por los propios colegas de trabajo, por los pacientes y sus familiares.

Las contribuciones de este estudio abarcan a los profesionales participantes y a la categoría de enfermería, a las instituciones laborales, así como a los usuarios de los servicios de salud, promoviendo una mirada holística sobre la incidencia de la violencia en el ambiente de trabajo, posibilitando la creación de medidas para disminuir la ocurrencia de los acontecimientos, para implantar protocolos y educación continua para que los profesionales sepan cómo reaccionar ante una violencia, además de alentarlos a registrar y denunciar a los agresores. Además, pueden estimular el desarrollo de investigaciones futuras sobre la temática y la creación de políticas públicas orientadas a la salud del trabajador.

Se señala que, a pesar de las diferentes realidades socioculturales evidenciadas en los estudios, la violencia en el ambiente laboral es evidenciada por los diferentes autores como un problema global de los servicios de salud, especialmente en los servicios hospitalarios, afectando a diferentes profesionales y asociado a varios factores.

Con relación a las limitaciones de esta investigación, se destaca el estudio transversal, que impide la generalización de los datos, así como la no investigación de los individuos alejados del trabajo. Además, el hecho de que los instrumentos utilizados sean autoadministrados y puestos a disposición de los profesionales vía medios de comunicación (*WhatsApp*, correo electrónico), pudiendo sufrir influencia del interés de los profesionales.

Las contribuciones del estudio para el avance científico engloban la posibilidad de la creación de futuras políticas públicas para la protección de los profesionales de enfermería, teniendo potencial para el mejoramiento del ambiente de trabajo, impactando directamente en la asistencia prestada y en las instituciones laborales.

## Conclusión

Ante lo expuesto, fue posible evidenciar la ocurrencia significativa de episodios de violencia en el trabajo en los últimos 12 meses, siendo la violencia verbal la más referida. Se constató que los profesionales de enfermería están propensos a sufrir violencia proveniente de los usuarios de los servicios de salud, por su contacto continuo, y también de los propios colegas de trabajo y jefes/superiores, aunque en una frecuencia menor.

La violencia en el trabajo está presente en el equipo de enfermería de forma asidua, lo que demuestra la importancia de políticas públicas para el mejoramiento de la seguridad en el trabajo, así como la capacitación y educación continua de los trabajadores de enfermería para que sepan reaccionar ante la variedad de abusos.

Se enfatiza, además, la necesidad de mejorar la estructura física de los servicios, el suministro de equipos necesarios y la adecuación de recursos humanos, además de ser de suma importancia el apoyo de los liderazgos y gestión de la salud, para que los profesionales se sientan seguros al denunciar los actos de violencia. Asimismo, se destaca la importancia de desmitificar el tema de la salud del trabajador entre los profesionales de enfermería, para que la subnotificación sea cada vez menor.

Además, se sugiere el estímulo a nuevas investigaciones sobre la violencia en el trabajo para que, de esta forma, sea posible incitar reflexiones para la reducción de los casos y un cambio de postura ante la ocurrencia del fenómeno.
